# Calculation of the Optimal Angiographic View Using Intravascular Ultrasound by the Tip Detection Method

**DOI:** 10.1016/j.jaccas.2025.104413

**Published:** 2025-07-30

**Authors:** Yohei Fukata, Ryohei Yoshikawa, Atsunori Okamura, Hideto Tawa, Yasuhiro Ueda, Naoya Yasuda, Daiji Kashiwagi, Kensuke Kondo, Masanobu Okamoto, Katsunori Wakayama

**Affiliations:** a Department of Clinical Engineering, Sanda City Hospital, Sanda, Hyogo, Japan; b Department of Cardiovascular Medicine, Sanda City Hospital, Sanda, Hyogo, Japan; c Cardiovascular Center, Sakurabashi Watanabe Advanced Healthcare Hospital, Osaka, Japan

**Keywords:** angiography, intravascular ultrasound, tip detection

## Abstract

**Objectives:**

We aimed to calculate the optimal angiographic view from an intravascular ultrasound (IVUS) image using the tip detection (TD) method.

**Key Steps:**

Using the TD method, we identified the orientation of the angiographic image on the IVUS image. The IVUS image was superimposed on the angiographic image. The angle at which the target could be most clearly distinguished was measured using the angle measurement function of the IVUS device. The position of the detector providing the optimal angiographic view was determined.

**Potential Pitfalls:**

The detector has restricted range of motion. Also, the direction of movement of the detector can be complicated where sharp curvature of the vessels occurs.

**Take-Home Messages:**

Our technique, “calculation of the optimal angiographic view using IVUS” (COA view), yielded good results for treating bifurcation lesions. The COA view method can be used to determine the optimal angiographic view from IVUS images.

## Visual Summary

**Figure:**
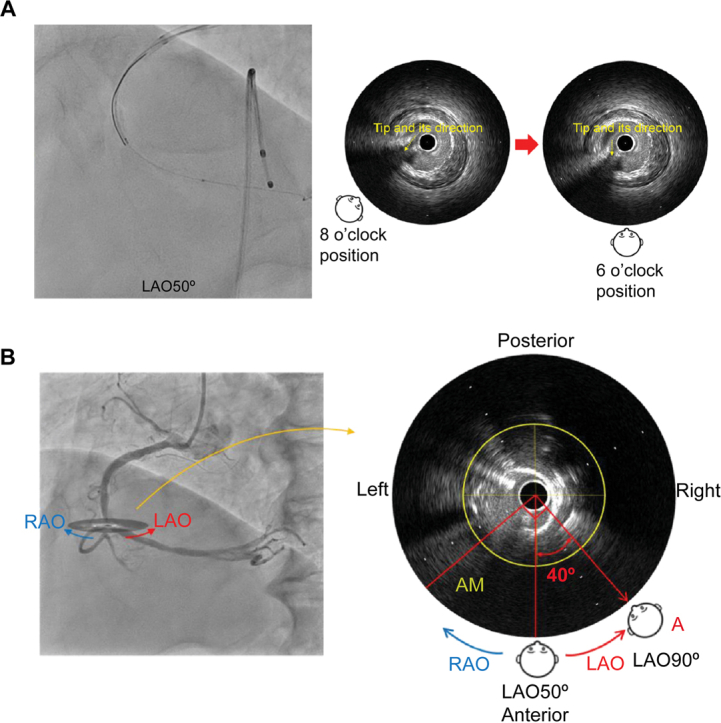

**Visual Summary:**
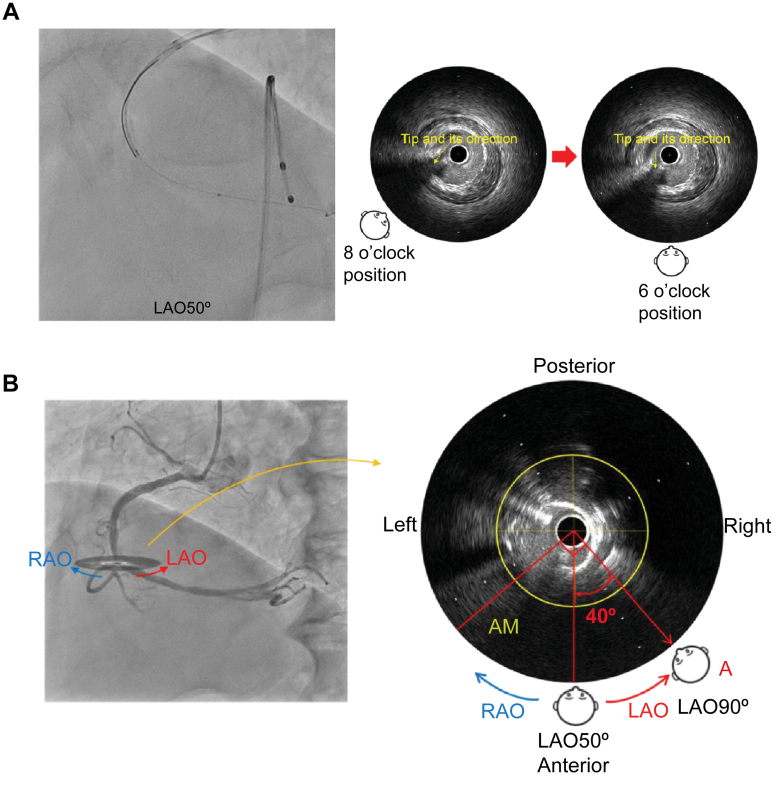
Calculation of the Optimal Angiographic View Using IVUS (A) To determine the angiographic view on intravascular ultrasound (IVUS), the tip of the guidewire is directed toward the detector, that is, toward the operator. By using the tip detection method to visualize the tip direction on IVUS, the operator's viewpoint is determined and set at the 6 o'clock direction. This alignment allows the IVUS image to be superimposed on the angiographic image. (B) The angle relative to the position perpendicular to the acute marginal (AM) branch on the IVUS image is 40° counterclockwise from the 6 o'clock direction. Therefore, the optimal angiographic view for clearly distinguishing the bifurcation is left anterior oblique (LAO) at 90°. RAO = right anterior oblique.

The tip detection (TD) method was devised to support wire manipulation for confirming the direction of the wire tip in real time by using an intravascular ultrasound (IVUS) system equipped with a pull-back function.^1^ This method is becoming indispensable as it supports the treatment of chronic total occlusion lesions. We actively use the TD method not only for treating chronic total occlusion lesions but also in routine percutaneous coronary intervention (PCI).

**Take-Home Messages**

• Using the COA view, we can calculate the optimal angiographic view for performing percutaneous coronary intervention, such as one in which the side branches are clearly distinguished.
• This approach facilitates guidewire manipulation, allowing the operator to perform the procedure more easily, accurately, and safely.

The IVUS image can be superimposed on an angiographic image using the TD method, enabling accurate determination of the target position on the angiographic image.^2^ We aimed to calculate the optimal angiographic view using IVUS images and provide operators with this information to enable them to perform the procedure more easily, accurately, and safely. We termed this technique “calculation of the optimal angiographic view using IVUS,” hereafter referred to as the “COA view.” We report an example of its real-world use.

## Case Summary

**Figure 1:**
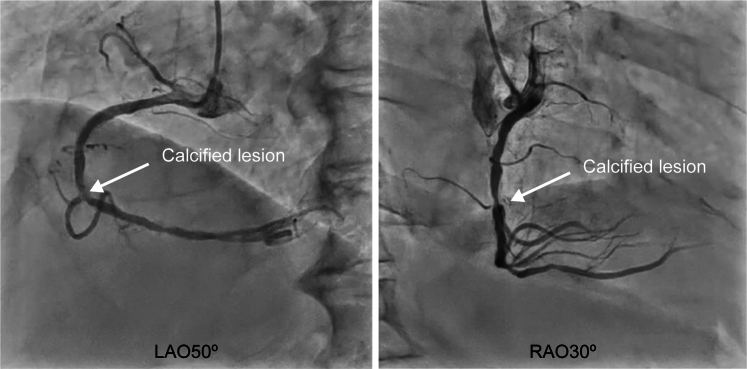
Initial Angiographic Image A calcified focal lesion is observed in segment 2 of the RCA. LAO = left anterior oblique; RAO = right anterior oblique.

An 81-year-old man underwent PCI to treat acute myocardial infarction. Balloon dilatation of the culprit lesion in segment 2 of the right coronary artery (RCA) was performed, and reperfusion was achieved; however, as dilatation was insufficient, the patient was scheduled to undergo a repeat PCI at our hospital at a later date, including debulking (Figure 1). Later, after the treatment, including debulking, was explained and written consent was obtained, PCI was performed. After using the Rotablator, predilatation was performed, and a stent was placed. Subsequent angiography revealed delayed contrast flow in the acute marginal (AM) branch, and a wire recrossing was performed. However, this was difficult in the left anterior oblique (LAO) at 50°, where the bifurcation was unclear. The COA view technique was used to calculate the angiographic view, which enabled a clear distinction of the bifurcation.

## Procedural Steps

### Step 1

A system similar to that used for the TD method was established.

**Figure 2:**
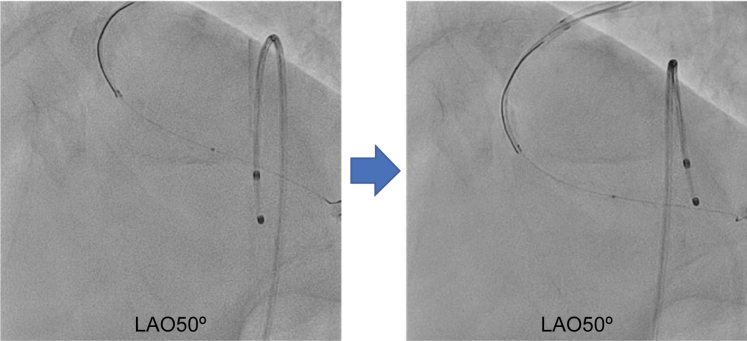
Angiographic Image With the Tip Pointing Toward the Detector Counterclockwise rotation of the image such that the tip and shaft are aligned in a straight line, which means that the tip is pointing toward the operator's viewpoint. LAO = left anterior oblique.

**Figure 3:**
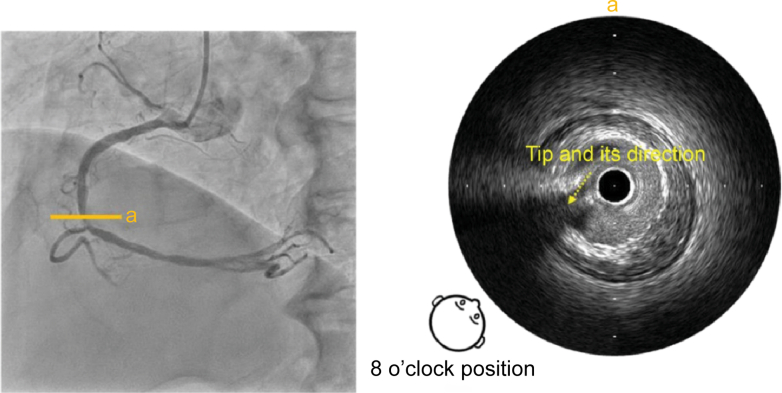
Identification of the Angiographic View on the IVUS Image IVUS image from point a. The angiographic view viewed by the operator (LAO at 50°) was observed from approximately the 8 o'clock position on the IVUS image. IVUS = intravascular ultrasound; LAO = left anterior oblique.

First, the IVUS catheter was inserted over the first guidewire, followed by the insertion of the second guidewire. To determine the angiographic view on IVUS, the tip of the guidewire was directed toward the detector, that is, toward the operator. When the tip of the second guidewire appeared to the left on fluoroscopy, it was rotated counterclockwise until the tip and shaft formed a straight line (Figure 2). Aligning the tip with the shaft in this manner eliminated discrepancies caused by visual perception, ensuring a clear orientation for the operator. At this point, the angiographic view was LAO at 50°. On IVUS, the tip was visualized, pointing approximately in the 8 o'clock direction, indicating that the angiographic view (LAO at 50°) observed by the operator corresponded to approximately the 8 o'clock position on the IVUS image (Figure 3).

### Step 2

The viewpoints were unified in line with the course of the blood vessels to make it easier to superimpose the IVUS image onto the angiographic image. If the vessel ran left to right, the IVUS image was rotated such that the operator's viewpoint was in the 3 o'clock direction, and if it ran up to down, the image was rotated such that the operator's viewpoint was in the 6 o'clock direction.^3^

**Figure 4:**
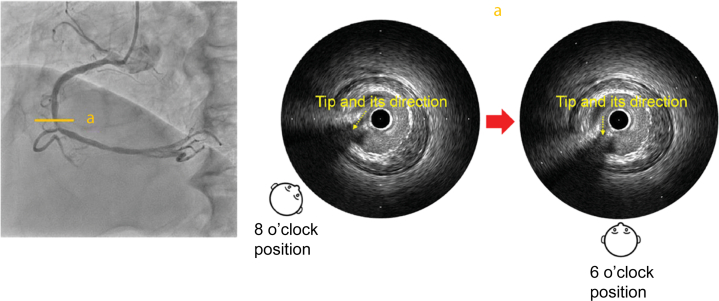
Unification of Viewpoints in Line With the Course of the Blood Vessel IVUS image from point a. Because the mid-RCA runs up to down, the IVUS image is rotated such that the operator's viewpoint is in the 6 o'clock direction with respect to the image. IVUS = intravascular ultrasound; RCA = right coronary artery.

Because the mid-RCA runs up to down, the IVUS image was rotated such that the operator's viewpoint was in the 6 o'clock direction with respect to the image (Figure 4).

On the IVUS image, the 6 o'clock direction was anterior, and the 12 o'clock direction was posterior from the operator's viewpoint, allowing the IVUS image to be superimposed on the angiographic image.

**Figure 5:**
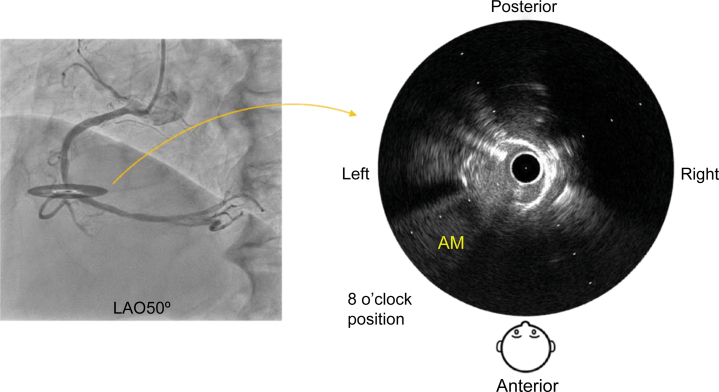
Bifurcation After “Fusion” On the IVUS image, the opening of the acute marginal branch is in approximately the 8 o'clock position, and on the angiographic image, it can be seen to open from the left anterior direction. AM = acute marginal; IVUS = intravascular ultrasound.

The AM branch opened at approximately the 8 o'clock position (Figure 5).

We termed the steps described thus far as “Fusion.”

### Step 3

The position perpendicular to the AM branches on the IVUS image appeared to be the most clearly distinguished on the angiographic image.

The angle at which the AM branch can be most clearly distinguished was measured using the angle measurement function of the IVUS device.

**Figure 6:**
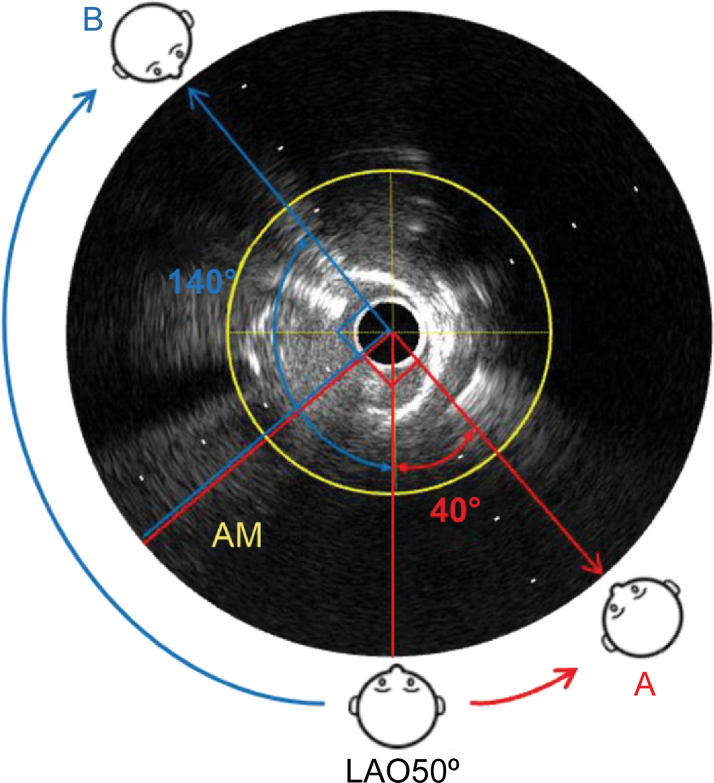
Measurement of the Angle Between the AM Branch and the Position Perpendicular to It The angle relative to the position perpendicular to the AM branch on the IVUS image is 40° counterclockwise from the 6 o'clock direction at point A and 140° clockwise at point B. AM = acute marginal; LAO = left anterior oblique.

The angle relative to the position perpendicular to the AM branch on the IVUS image was 40° counterclockwise from the 6 o'clock direction at point A and 140° clockwise at point B, and the measurement was performed using the center of the vessel as the midpoint (Figure 6). From the IVUS images, we could determine the detector direction in which the AM branch could be most clearly distinguished in the current angiographic view (LAO at 50°).

### Step 4

The position of the detector providing the optimal angiographic view was considered.

In theory, because the IVUS image is cross-sectionally perpendicular to the long axis of the blood vessel, the direction of movement of the detector is around circumference of the blood vessel. However, in clinical practice, the approximate direction of detector movement can be calculated by combining the orientation perpendicular to the long axis of the vessel with the direction in which the vessel depicted on IVUS appears to be the most stretched on angiographic images.

The detector movement directions for the main segments are as follows:^3^

• Mid-RCA: right anterior oblique (RAO) 90° to LAO 90°.
• Distal RCA: LAO 45° cranial (CRA) 45° to LAO 45° caudal (CAU) 45°.
• Proximal left anterior descending artery: RAO 30° CRA 45° to RAO 30° CAU 45°.
• Mid–left anterior descending artery: RAO 45° CRA 30° to LAO 45° CRA 30°.
• Proximal left circumflex artery: LAO 45° CRA 45° to LAO 45° CAU 45°.
• Mid–left circumflex artery: LAO 45°to CAU 30°.

For the mid-RCA, on the angiographic image, the LAO is on the right and the RAO is on the left; on the IVUS image, LAO is counterclockwise and RAO is clockwise.

**Figure 7:**
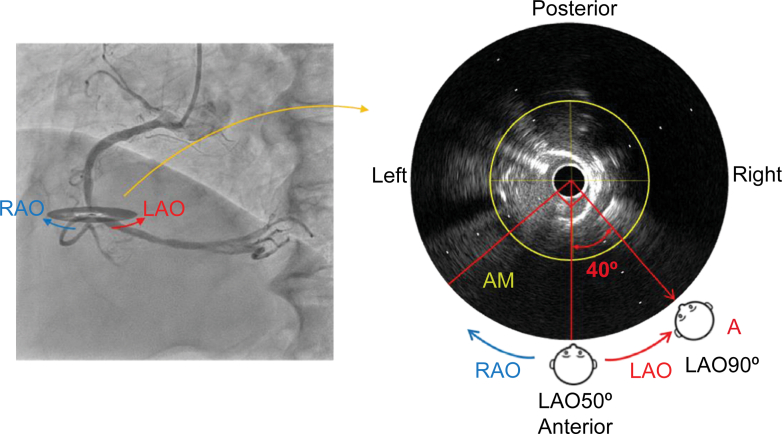
Determination of the Detector Position Enabling the Bifurcation to Be Most Clearly Distinguished On the angiographic image, right is LAO and left is RAO. On the IVUS image, counterclockwise is LAO and clockwise is RAO. The optimal angiographic view selected is LAO at 90°. AM = acute marginal; IVUS = intravascular ultrasound; LAO = left anterior oblique; RAO = right anterior oblique.

Therefore, in this case, the optimal angiographic view that can most clearly distinguish the bifurcation is LAO at 90°, which is achieved by moving 40° in the LAO direction, or RAO at 90°, which is achieved by moving 140° in the RAO direction. We selected LAO at 90° (Figure 7). In clinical practice, it is beneficial to consider the direction of movement within the range in which the detector can move. We termed the steps described thus far the “COA view” technique.

**Figure 8:**
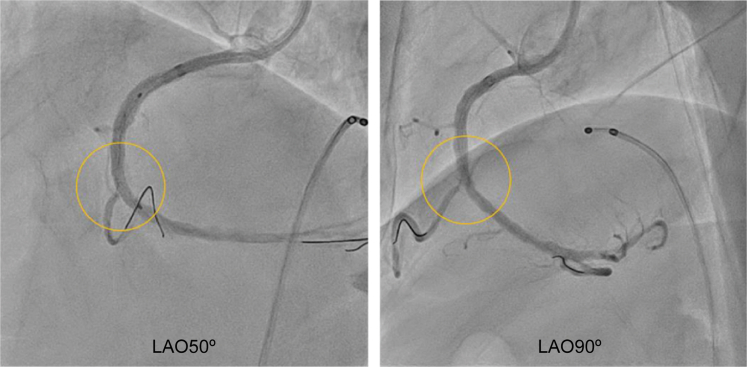
Comparison of Angiographic Images of the Bifurcation The bifurcation could be more clearly distinguished from LAO at 90° than from LAO at 50°. LAO = left anterior oblique.

The bifurcation could be clearly distinguished (Figure 8).

**Figure 9:**
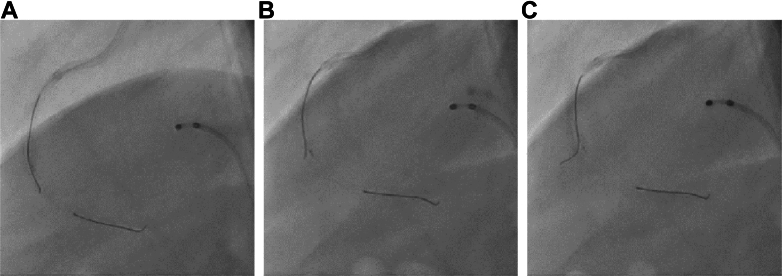
Wiring After the COA View Technique The tip of the guidewire is pointed left at a position slightly beyond the bifurcation (A). It is gradually withdrawn, and when the tip enters the acute marginal branch, its behavior changes (B). The guidewire is advanced in this position (C). COA view, calculation of the optimal angiographic view using intravascular ultrasound. COA view = calculation of the optimal angiographic view using intravascular ultrasound.

The COA view technique was successfully performed. We then performed wiring in the AM branch. The IVUS catheter was placed in the bifurcation; the tip of the second guidewire was pointed left, at a position slightly beyond the bifurcation (Figure 9A), and slowly withdrawn. When the tip of the guidewire entered the AM branch, its behavior changed (Figure 9B); by advancing the guidewire in this position, recrossing was successfully achieved (Figure 9C). The procedure was completed without losing the AM branch.

## Potential Pitfalls

The detector has a restricted range of motion, and depending on the calculation results, bifurcation cannot be clearly distinguished in some cases. However, fusion enables an accurate assessment of the direction of bifurcation, which is important information for the operator.

Furthermore, in places of sharp curvature of the vessels, the direction of movement of the detector was slightly complicated. Therefore, it should be used within clinically acceptable limits.

## Conclusions

In this case, wire crossing of the AM branch proved impossible to achieve with the routine angiographic view of LAO at 50°, but using the COA view technique, we were able to clearly distinguish the bifurcation and obtain an accurate grasp of its morphology—information extremely important for performing wire crossing. We positioned the guidewire slightly beyond the bifurcation with the tip pointing toward the bifurcation, which had become clear, and withdrew it gradually. This made recrossing comparatively easy, and such a technique may help with completing distal stent strut procedures.

After recrossing, the recrossing point can be checked using IVUS. If recrossing is unsuccessful, the TD method can be attempted.

## Funding Support and Author Disclosures

The authors have reported that they have no relationships relevant to the contents of this paper to disclose.
